# A TLR9 agonist enhances the anti-tumor immunity of peptide and lipopeptide vaccines via different mechanisms

**DOI:** 10.1038/srep12578

**Published:** 2015-07-28

**Authors:** Ying-Chyi Song, Shih-Jen Liu

**Affiliations:** 1 National Institute of Infectious Diseases and Vaccinology, National Health Research Institutes, Miaoli, Taiwan; 2 Research Center for Chinese Medicine & Acupuncture, China Medical University, Taichung, Taiwan; 3 Graduate Institute of Immunology, China Medical University, Taichung, Taiwan

## Abstract

The toll-like receptor 9 (TLR9) agonists CpG oligodeoxynucleotides (CpG ODNs) have been recognized as promising adjuvants for vaccines against infectious diseases and cancer. However, the role of TLR9 signaling in the regulation of antigen uptake and presentation is not well understood. Therefore, to investigate the effects of TLR9 signaling, this study used synthetic peptides (IDG) and lipopeptides (lipoIDG), which are internalized by dendritic cells (DCs) via endocytosis-dependent and endocytosis-independent pathways, respectively. Our data demonstrated that the internalization of lipoIDG and IDG by bone marrow-derived dendritic cells (BMDCs) was not enhanced in the presence of CpG ODNs; however, CpG ODNs prolonged the co-localization of IDG with CpG ODNs in early endosomes. Surprisingly, CpG ODNs enhanced CD8^+^ T cell responses, and the anti-tumor effects of IDG immunization were stronger than those of lipoIDG immunization. LipoIDG admixed with CpG ODNs induced low levels of CD8^+^ T cells and partially inhibit tumor growth. Our findings suggest that CpG ODNs increase the retention of antigens in early endosomes, which is important for eliciting anti-tumor immunity. These results will facilitate the application of CpG adjuvants in the design of different vaccines.

Peptide-based vaccines have several advantages over conventional whole-protein vaccines in terms of purity, lot-to-lot consistency, production costs, and the high specificity of the elicited immune responses^1^,^2^,^3^. However, the use of peptide antigens in vaccine development has been hampered by problems, such as weak immunogenicity coupled with a paucity of sufficiently potent adjuvants that can be tolerated by humans. However, studies have demonstrated that certain modifications, such as increasing the length of peptides or conjugating peptides to palmitic acid lipid tails, significantly improves their immunogenicity^4^,^5^. Our previous study demonstrated that synthetic long peptides, which contained the CTL epitope of HPV16 E7 and were modified with mono-palmitic acids, elicited stronger CTL responses and antitumor effects than their non-lipidated counterparts. Moreover, the lipidated long peptides were internalized and cross-presented by bone marrow-derived dendritic cells (BMDCs) via an endocytosis-independent mechanism. In contrast, non-lipidated peptides are internalized via an endocytosis-dependent mechanism^6^. The induction of CTL responses by lipidated and non-lipidated long peptide vaccines may be enhanced when these vaccines are formulated with incomplete Freud’s adjuvant (IFA). Therefore, the selection of suitable adjuvants for peptide-based vaccines is critical to enhance CTL responses against virus-infected cells and cancer cells.

More than 100 adjuvant preparations have been described^7^; however, only aluminum hydroxide and aluminum phosphate adjuvants are widely used in human vaccines. However, the principal limitation of aluminum salts is their inability to elicit CTL responses^8^, which is an important protective mechanism against microbial infections and tumors. The vertebrate innate immune system has developed various strategies to detect pathogens based on the recognition of certain signature molecules of pathogens. Oligonucleotides that contain unmethylated CpG dinucleotides (CpG ODNs), a characteristic of bacterial DNA, are detected by TLR9 and serve as a ‘danger signal’ for the innate immune system, thereby triggering a protective immune response. Similarly, synthetic CpG ODNs can stimulate B cells, monocytes, macrophages and DCs. In addition, CpG ODNs can induce the secretion of pro-inflammatory cytokines (IL-1, IL-6, IL-18 and TNF-α) and type 1 cytokines (IL-12, IFN-γ), which results in the generation of a helper T cell type 1 (Th1)-biased immune response^9^,^10^,^11^. In humans, CpG ODNs have been used as adjuvants for infectious diseases either alone or combination with alum^12^,^13^. Many studies have found that mixtures of protein antigens and CpG ODNs have substantial effects, such as tumor rejection and heightened Th1-biased immunity^14^,^15^. Furthermore, the combination of various TLR agonists with antigens may induce variably effective T cell responses and antiviral protection in mice^16^. However, the effector mechanisms of TLR9 agonists in antigen presentation by major histocompatibility complex (MHC) class I molecules remain unclear.

The co-administration of antigen and CpG ODNs is important for targeting the same antigen-presenting cells (APCs)^17^. In a previous study, CpG ODNs were subsequently administered following the administration of antigen; however, antigen-specific immune responses were not enhanced^18^. This finding led many studies to focus on the co-delivery of antigen and CpG ODNs to antigen-presenting cells (APCs) for the induction of cellular immunity. However, a limited number of studies have investigated the uptake of antigen by APCs via the co-administration of antigen and CpG ODNs. Immature DCs take up antigen more efficiently than mature DCs but have a poorer antigen presentation ability^19^. The maturation and activation of DCs may be triggered by the TLR9 ligand, which inspired our efforts to use TLR9 ligand-stimulated DCs against infectious diseases and cancer^20^,^21^,^22^. Exogenous antigen uptake by DCs depends on the size and nature of the antigen^23^,^24^; however, the presence of TLR ligands may regulate the uptake process and further enhance cross-presentation efficacy. The cross-presentation of exogenous antigens by DCs is regulated by the engagement of TLRs and cytokines^25^; however, it remains unclear whether the co-administration of different types of antigens with TLR ligands can increase the antigen uptake ability or antigen presentation efficiency of DCs. In contrast, synthetic cationic peptides modulate the uptake of CpG ODNs, which enhances their immunostimulatory effects^26^,^27^. These results suggest that the interaction of antigens and CpG ODNs may have significant effects on the activation of APCs and antigen processing.

Our previous study demonstrated that lipidated long peptide antigens in an incomplete IFA formulation enhanced peptide-specific T cell effects and antitumor responses compared with non-lipidated long peptide antigens^6^. In this study, we investigated the role of CpG ODNs as adjuvants to enhance non-lipidated long peptide- or lipidated long peptide-induced anti-tumor immunity. We found that the non-lipidated long peptides induced strong CTL responses and anti-tumor effects when combined with CpG ODNs. Surprisingly, CpG ODNs only moderately enhanced the CTL responses and anti-tumor effects when combined with the lipidated long peptide. These findings suggest that CpG ODNs may enhance anti-tumor immunity via different mechanisms.

## Results

### CpG ODNs have different effects on the intracellular trafficking of peptides and lipopeptides

**Figure 1:**
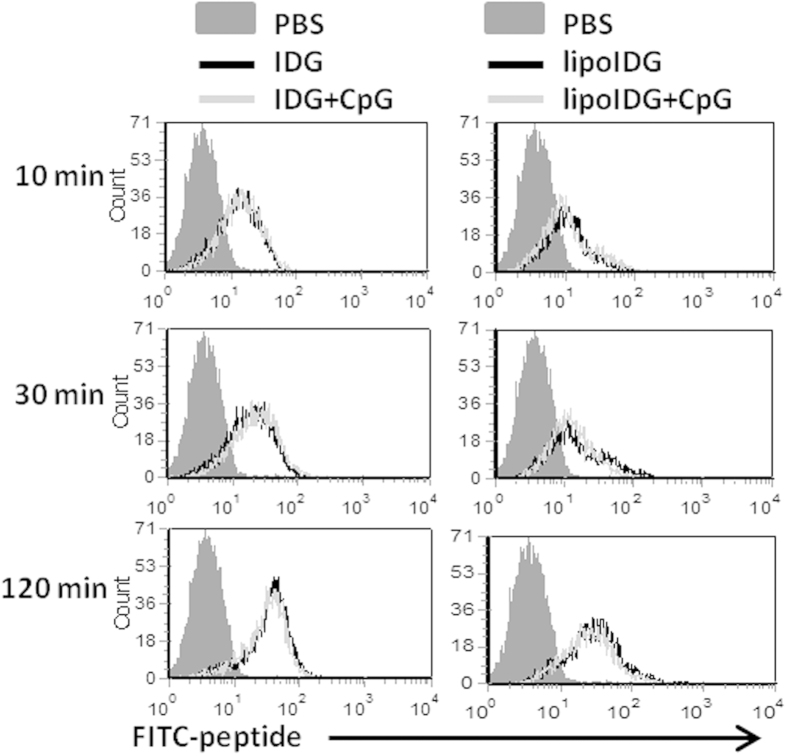
Internalization efficiency of the peptides and lipopeptides with or without CpG ODNs. The cellular internalization activities at different time points were determined by flow cytometry after DCs were treated with FITC-conjugated IDG or lipoIDG (1 μg/ml) (green) combined with or without CpG ODNs (10 μg/ml). FITC-conjugated IDG or lipoIDG combined with or without CpG ODNs was incubated with BMDCs for 10, 30 and 120 min. After trypan blue quenching, the internalization of the peptides and lipopeptides by CD11c^+^ cells was analyzed using flow cytometry. Dead cells were gated out by propidium iodide staining. These data are representative of three independent experiments.

**Figure 2:**
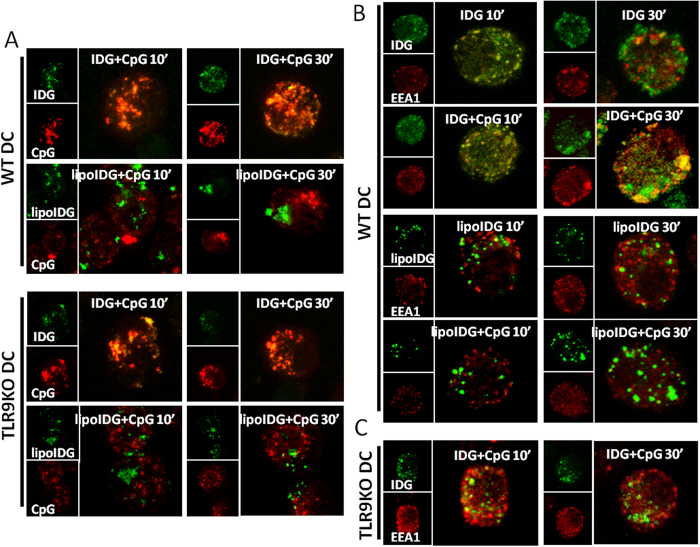
Intracellular trafficking of the peptides and the lipopeptides induced by CpG ODNs. Intracellular trafficking at different time points was determined by confocal microscopy. (**A**) Wild-type and TLR9 knockout BMDCs (WT DCs and TLR9 KO DCs) were treated with FITC-conjugated IDG or lipoIDG (5 μg/ml) (green) combined with Cy5-conjugated CpG ODNs (10 μg/ml) (red) for 10 and 30 min. After incubation, the cells were visualized by confocal microscopy. (**B**) WT DCs and (**C**) TLR9 KO DCs were pulsed with FITC-conjugated IDG or lipoIDG (green) for 10 and 30 min. After incubation, the cells were stained with an anti-EEA1 Ab (red) and visualized by confocal microscopy. IDG and lipoIDG are indicated in green, and CpG and EEA1 are shown in red (the left panel of each group). Merged colors are shown in yellow (the right panel of each group). These data are representative of three independent experiments.

 Antigen capture is the first step required for cross-presentation, and antigen uptake mechanisms are closely linked with the intracellular trafficking and fate of the antigen^28^. To determine the internalization efficiency of the peptides and lipopeptides combined with or without CpG ODNs in BMDCs, FITC-conjugated IDG or lipoIDG was incubated with BMDCs in the absence or presence of CpG ODNs for 10, 30 and 120 min. The internalization of the FITC-conjugated peptides and lipopeptides was analyzed by flow cytometry after trypan blue was used to quench surface-associated fluorescence. As shown in Fig. 1, both IDG and lipoIDG were efficiently internalized by BMDCs in as early as 10 min. However, the internalization was not enhanced in the presence of CpG ODNs (Fig. 1). In addition, we found that FITC-conjugated RAH (specific CTL epitope derived from HPV16 E7 protein) could not be internalized in BMDCs even in the presence of CpG ODNs (data not shown). Subsequently, CpG ODNs are taken up and trafficked to endosomes via nonspecific endocytosis or receptor-mediated endocytosis^29^,^30^,^31^. To determine whether the internalization of IDG, lipoIDG and CpG ODNs by BMDCs similarly occurred, FITC-conjugated IDG and lipoIDG combined with Cy5-conjugated CpG ODNs were incubated with BMDCs for 10 and 30 min. We found that intracellular IDG, but not lipoIDG, efficiently colocalized with CpG ODNs in both wild-type (WT) and TLR9 knockout (KO) BMDCs (Fig. 2A). Our previous study demonstrated that IDG, but not lipoIDG, was internalized by BMDCs and was detected in early endosomes^6^. To further determine whether the intracellular localization of the peptides and lipopeptides could be regulated by CpG ODNs, BMDCs were incubated with FITC-conjugated IDG or lipoIDG in the absence or presence of CpG ODNs. Then, an antibody (Ab) against early endosome antigen-1 (EEA1) was used to detect early endosomes. After the incubation of IDG alone with BMDCs, internalized IDG was detected in early endosomes at 10 min but decreased at 30 min. By contrast, most of internalized IDG could be detected at 30 min in the presence of CpG ODNs (Fig. 2B). However, lipoIDG was not localized in early endosomes, and the presence of CpG ODNs did not change the localization (Fig. 2B). To confirm the retention of IDG in early endosomes in the presence of CpG ODNs, BMDCs that were derived from TLR9 KO mice were used. We found that the retention of IDG in early endosomes was reversed in TLR9 KO BMDCs (Fig. 2C). Taken together, these findings suggest that CpG ODNs did not facilitate the internalization of peptide and lipopeptide antigens in BMDCs but increased the retention of peptides in early endosomes. The divergent effects of CpG ODNs on peptides and lipopeptides may lead to different adjuvant functions.

### The peptides and lipopeptides did not enhance the CpG ODN-induced activation of BMDCs

**Figure 3:**
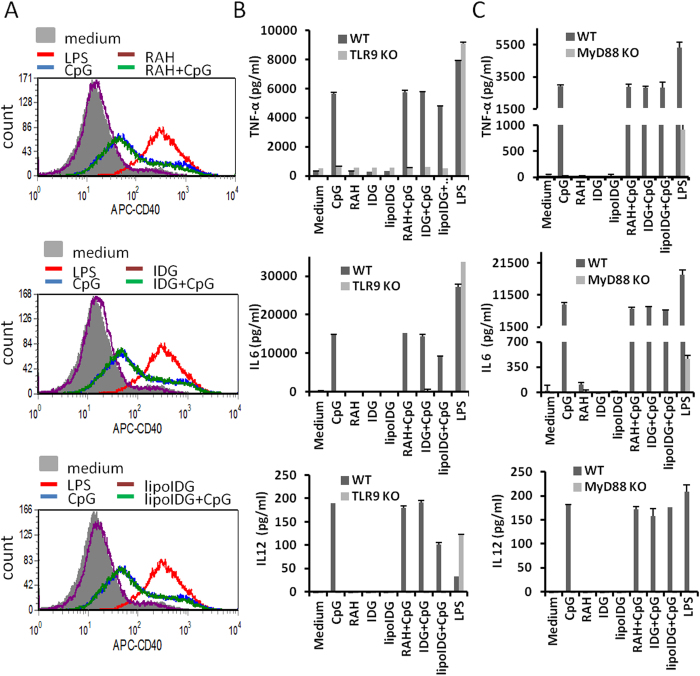
CpG ODN adjuvant induces DC maturation. Wild-type (WT) DCs, TLR9 knockout (TLR9 KO) DCs or MyD88 knockout (MyD88 KO) DCs were treated with RAH, IDG or lipoIDG (1 μM) combined with or without CpG ODNs (1 μM) for 18 h. LPS (0.1 μg/ml) was used as a positive control. (**A**) The maturation of DCs (gated on CD11c^+^ cells) was measured by staining with APC-labeled CD40 Ab and analyzed by flow cytometry. The levels of TNF-α, IL-6 and IL-12p70 in the culture supernatants from TLR9 KO DCs (**B**) and MyD88 KO DCs (**C**) were measured by ELISA. The results are expressed as the mean + S.D. of the amount of each cytokine.

Our previous data indicated that mono-palmitoylated long peptides (lipoIDG) and non-lipidated long peptides (IDG) were taken up by DCs using endocytosis-independent and endocytosis-dependent pathways, respectively. Therefore, we asked whether the co-administration of peptides that are internalized via different mechanisms with CpG ODNs would affect TLR9 ligation-induced DC activation. In this study, BMDCs were incubated with peptides alone or with peptides mixed with CpG ODNs for 18 h. The data revealed that the peptides (RAH, IDG or lipoIDG) alone did not induce the up-regulation of the co-stimulatory molecule CD40. The co-administration of the peptides with CpG ODNs did not enhance the up-regulation of CD40 compared with CpG ODNs administered alone (Fig. 3A). Accordingly, cytokine (TNF-α, IL-6 and IL-12p70) production was induced by CpG ODNs when administered alone; however, no significant differences were observed in the presence of the peptides (Fig. 3B,C). Furthermore, CpG ODN-induced cytokine production was dependent on the TLR9 and MyD88 pathway, because the activation effect was not observed when the BMDCs from TLR9 and MyD88 KO mice were treated with CpG ODNs (Fig. 3B,C). These observations suggest that the co-administration of CpG ODNs with different peptides that are internalized into APCs by different mechanisms did not affect TLR9-induced DC activation.

### CpG ODNs dramatically enhance peptide-induced, but not lipopeptide-induced, CTL responses

**Figure 4:**
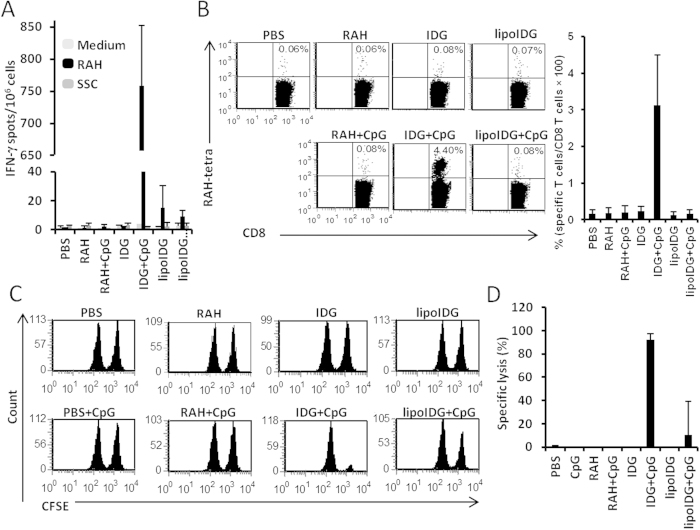
The effects of CpG ODN adjuvant on specific T cell responses. The C57BL/6 mice (2 animals/group) received a single injection of 1 μg of RAH, IDG or lipoIDG admixed with or without CpG ODNs (10 μg). (**A**) After 7 days, splenocytes were stimulated with the RAH peptide or an irrelevant peptide (SSC) (10 μg/ml) and examined for IFN-γ-secreting cells by ELISPOT. The data are expressed as the mean + S.D. of two animals per group. (**B**) The splenocytes were isolated from the immunized mice at 7 days and were stained with an FITC-CD4 Ab, a PE-conjugated RAH-tetramer, a Per-CP-CD8 Ab and an APC-CD19 Ab. The gated CD4^−^ and CD19^−^ populations were analyzed for the number of RAH-tetramer^+^ and CD8^+^ cells using flow cytometry. The number within each panel represents the percentage of the specific CD8^+^ T cells (up quadrants) among the total population of CD8^+^ T cells. The data in the right panel were expressed as the mean + S.D. of the specific CD8^+^ T cell percentages from four animals per group. (**C**) Naïve splenocytes were pulsed with the RAH peptide or an irrelevant peptide and stained for 15 minutes at 37 °C with 10 μM or 1 μM of CFSE, respectively. Both types of CFSE-labeled cells were injected into the vaccinated mice, and the spleens were removed after 18 h and analyzed by flow cytometry. (**D**) The specific lysis percentages are expressed as the mean + SEM of four animals per group. The following equation was used to determine specific lysis: % Specific lysis = [(% irrelevant peptide (low CSFE) × AF) – % RAH peptide (high CFSE)]/(% irrelevant peptide × AF). The experiment was repeated twice. Adjustment factor (AF) = % RAH peptide (high CSFE)/% irrelevant peptide (low CFSE) from the naïve controls.

Previous studies have found that mixtures of protein antigens and CpG ODNs have substantial effects, such as tumor rejection and heightened Th1-biased immunity^14^,^15^. Therefore, we assessed whether the prolonged retention time of IDG in endosomes, which was induced by CpG ODN, could affect the efficiency of cross-priming to CD8^+^ T cells. The mice were injected subcutaneously (s.c.) once with PBS, IDG or lipoIDG in the presence or absence of CpG ODNs. After 7 days, splenocytes were collected and stimulated with the RAH peptide or an irrelevant peptide to detect IFN-γ-secreting cells in an ELISPOT assay. Surprisingly, we found that the IFN-γ-secreting CD8^+^ T cells were significantly increased when IDG, but not lipoIDG, was combined with CpG ODNs (Fig. 4A). To further confirm the observation that CpG ODNs enhance antigen-specific CD8^+^ T cells *in vivo*, the splenocytes from the immunized mice were stained with a PE-conjugated RAH-tetramer. The data indicated that the co-administration of IDG and CpG ODNs resulted in increased numbers of antigen-specific CD8^+^ T cells compared with the co-administration of lipoIDG and CpG ODNs (Fig. 4B). These data suggest that CpG ODNs can enhance IDG vaccination-induced CD8^+^ T responses in contrast to lipoIDG vaccination. We further investigated whether the induced CD8^+^ T cells were capable of killing cells *in vivo*. The mice were immunized with PBS, RAH, IDG or lipoIDG in the presence or absence of CpG ODNs. After 7 days, 5-carboxyfluorescein diacetate succinimidyl ester (CFSE)-labeled target cells were adoptively transferred to the immunized mice for 18 h. A large amount of peptide-pulsed cells were killed in the mice that were immunized with IDG and CpG ODNs (IDG + CpG). However, a mild killing effect was observed in the mice that were immunized with lipoIDG and CpG ODNs (lipoIDG + CpG). The peptides alone (RAH, IDG, and lipoIDG) could not kill the target cells in the immunized mice. Notably, the mice that were immunized with RAH and CpG ODNs (RAH + CpG) did not exhibit any killing ability (Fig. 4C). The specific lysis percentages were 0%, 92.1 ± 5% and 10.5 ± 28.5% in the RAH + CpG, IDG + CpG and lipoIDG + CpG groups, respectively (Fig. 4D). Because antigen processing and presentation is necessary to prime CTL responses using IDG and lipoIDG, but not RAH, CpG ODNs may be involved in the regulation of antigen processing and presentation. These results indicate that CpG ODNs increased the processing and presentation of non-lipidated peptide antigen.

### CpG ODNs combined with peptides induce strong anti-tumor responses via TLR9

**Figure 5:**
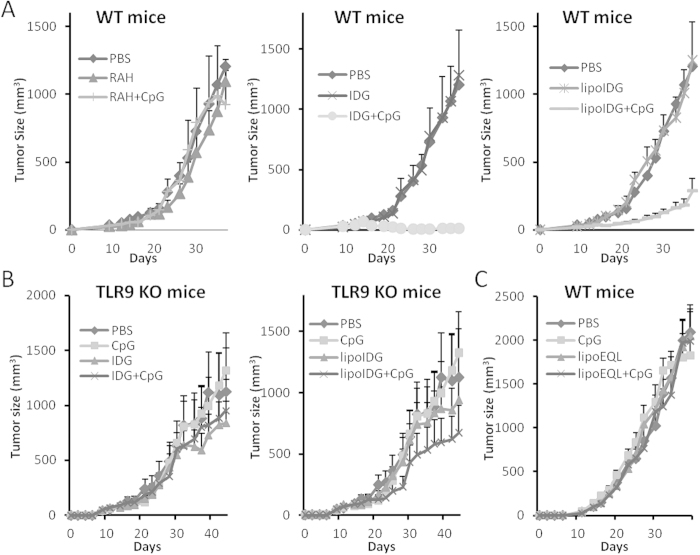
CpG ODN adjuvant induces anti-tumor effects, which are reversed in TLR9 KO mice. TC-1 tumor-bearing wild-type (WT) mice (**A**) and TC-1 tumor-bearing TLR9 knockout (KO) mice (**B**) (6 animals/group) received a single injection with RAH, IDG or lipoIDG (1 μg) mixed with or without CpG ODNs (10 μg). (**C**) The TC-1 tumor-bearing WT mice (6 animals/group) received a single injection with 1 μg of lipoEQL mixed with or without CpG ODNs (10 μg). PBS or CpG alone was used as a control. The tumor diameters are shown (mm^3^). The data are expressed as the means + SEM.

To determine whether CpG ODNs combined with peptides or lipopeptides could induce anti-tumor responses, TC-1 tumor-bearing mice were used. Seven days after the TC-1 cell inoculations, the mice were s.c. injected with peptides in the presence or absence of CpG ODNs. As shown in Fig. 5A, none of the mice that were immunized with PBS or peptide alone exhibited tumor growth inhibition. However, the mice that were immunized with lipoIDG + CpG displayed significantly delayed tumor growth. Furthermore, the mice that were immunized with IDG + CpG displayed completely inhibited tumor growth (Fig. 5A). To determine whether TLR9 engagement is necessary for tumor growth inhibition, tumor-bearing TLR9 KO mice were treated with IDG + CpG or lipoIDG + CpG. Figure 5B shows that the anti-tumor effects of IDG + CpG and lipoIDG + CpG were lost. In addition, the anti-tumor response was lost with IDG + CpG and lipoIDG + CpG vaccination in TC-1 tumor-bearing MyD88 knockout mice (Supplementary Fig. 1). Furthermore, we detected the Ag-specific CD8^+^ T cells and myeloid-derived suppressive cells (MDSCs) at the tumor site after different treatments. We found that the Ag-specific CD8^+^ T cell numbers were slightly higher in the IDG + CpG-treated group than in the other groups (Supplementary Fig. 2A). Interestingly, tumor-bearing mice immunized with IDG + CpG exhibited a significantly decreased number of MDSCs (Supplementary Fig. 2B). Therefore, the combination of IDG and CpG leads to the suppression of immunosuppressive cells in the local tumor environment and induces strong anti-tumor immunity. These results demonstrate that immunization with IDG combined with CpG ODNs provided robust and durable CTL responses and elicited superior anti-tumor activity compared with lipoIDG.

LipoIDG + CpG ODN induced strong anti-tumor effects; however, lipoIDG + CpG ODNs did not increase antigen-specific T cell responses and induced only low levels of CTL activity (Fig. 4). We speculated that lipoIDG + CpG may induce non-specific anti-tumor immunity. Therefore, we synthesized lipidated long peptides that were derived from OVA_aa_ (lipoEQL), which contained an H-2K^b^-restricted CTL epitope (SII peptide), to treat TC-1 tumor-bearing mice. As shown in Fig. 5C, TC-1 tumor growth was not inhibited in the lipoEQL + CpG group. These data demonstrated that the IDG + CpG immunization induced dramatic antigen-specific CTL responses and anti-tumor immunity. However, the lipoIDG + CpG immunization induced mild CTL responses and unknown anti-tumor immunity.

## Discussion

The cross-presentation of exogenous antigens by APCs on MHC class I molecules is crucial for the development of CD8^+^ CTL responses against tumors and infectious pathogens^32^,^33^. DCs are now known to be the most potent APCs and can cross-present exogenous antigens to stimulate T cells after being internalized during macropinocytosis, phagocytosis, or receptor-mediated endocytosis^19^. Evidence has indicated that TLR9 ligands increase the antigen cross-presentation activities of B cells, conventional DCs and plasmacytoid DCs^34^,^35^,^36^,^37^. However, the detailed regulatory mechanisms that underlie this effect are not well understood. In this study, we found that the TLR9 ligands CpG ODNs enhance endocytosis-dependent antigen cross-presentation (via a vacuolar pathway), but not endocytosis-independent antigen cross-presentation (via a cytosolic pathway), and increase endocytosis-dependent antigen retention in early endosomes. To confirm our observations in TLR9-rich plasmacytoid DCs (pDCs), we adoptively transferred IDG + CpG-pulsed BMDCs and IDG + CpG-pulsed Flt3L-cultured DCs (pDCs) into tumor-bearing mice. We found that the cross-presentation efficiency and the anti-tumor effects did not vary between the IDG + CpG-treated BMDCs and the pDCs (Supplementary Fig. 3).

Besides TLR9 ligand, other TLR ligands, such as the TLR2 ligand (lipopeptide)^38^,^39^, the TLR4 ligand (LPS)^36^,^40^,^41^, the TLR3 ligand (polyinosinic-polycytidylic acid)^42^, and the TLR7 ligand (R848, synthetic RNA oligonucleotides, or polyU)^43^,^44^,^45^, enhance DC activation and exogenous antigen cross-presentation. In addition, these TLR ligands play roles in the regulation of the antigen cross-presentation process in different APCs^34^,^44^,^45^. TLR4 agonists enhance antigen cross-presentation by increasing antigen internalization and delivery to the cytosol^40^. Moreover, TLR3, TLR7 and TLR9 agonists enhance TAP-dependent cross-presentation^34^,^45^. Furthermore, our previous study demonstrated that TLR2 agonist-conjugated long peptides (Pam2IDG) can enhance antigen cross-presentation via a TLR2-dependent and TAP-independent pathway^39^. These results suggest that different TLR agonists may enhance antigen cross-presentation via different mechanisms of intracellular antigen processing or delivery.

In contrast to TLR2 and TLR4, which recognize invariant foreign bacterial and viral constituents at the cell membrane, TLR9 is endosomally expressed in innate immune cells and recognizes distinct patterns of nucleic acids at endosomal compartments^29^,^46^. The mechanisms of CpG ODN uptake by APCs are controversial. Lahoud *et al.* found that DEC-205, a cell surface receptor for CpG ODNs, enhanced CpG ODN internalization^30^. Moseman *et al.* further demonstrated that mannose receptor 1 (CD206) is involved in endosomal delivery and the trafficking of CpG ODNs^47^. According to the present study, the uptaken CpG ODNs are colocalized with IDG in both WT DCs and TLR9 KO DCs (Fig. 2A). In addition, the long antimicrobial peptides LL-37 and KLKL_5_KLK have been demonstrated to facilitate the uptake of CpG ODNs and to enhance anti-tumor immunity^26^,^27^. Similar to the KLKL_5_KLK peptide, lipoIDG interacts with the membrane and is internalized by non-phagocytic cells^6^. However, neither lipoIDG nor IDG enhanced the uptake of CpG ODNs (Supplementary Fig. 4) or the activation of DCs (Fig. 3) in this study. Furthermore, early or recycling endosomes have been linked to signaling by TLR9^48^, and MHC class I was located in the recycling endosome compartment^49^. These studies suggested that MHC class I may access exogenous antigen and translocate to the cell surface after TLR9 activation. Therefore, we speculated that TLR9 signaling-induced endosome recycling may increase the interaction between the antigen and MHC class I molecules, resulting in IDG retention in the endosome, thereby eliciting strong anti-tumor immunity.

APCs internalize antigens and generate peptides via lysosomal proteases. In contrast to macrophages, which contain high levels of lysosomal proteases and rapidly degrade internalized antigens, DCs are protease-poor and therefore efficiently accumulate, process, and disseminate antigens^50^. In this study, we directly observed that intracellular CpG ODNs efficiently colocalize with IDG according to the confocal microscopy analysis (Fig. 2A). In addition, CpG ODNs prolonged the retention of IDG in early endosomes (Fig. 2B). The mechanisms of cellular trafficking may play an important role in CpG ODN-induced CD8^+^ T cell responses. In contrast, the different mechanisms of cellular trafficking may have resulted in the limited effects of lipoIDG immunization on CpG ODN-enhanced CTL responses that were observed in this study. Moreover, certain antigens led to the generation of peptides and to the cross-presentation of peptide-loading complexes by cathepsin S-dependent and TAP-independent pathways in endocytic compartments^39^,^51^. We speculated that this increased retention may increase the cross-presentation of antigens to MHC class I molecules, thereby eliciting strong anti-tumor immunity. Similar observations have been reported, which suggests that prolonged antigen survival and retention in early endosomes is important for cross-priming CTLs^52^,^53^. The detailed mechanisms by which CpG ODNs regulate antigen retention in early endosomes require further study. These results suggest that CpG ODNs may not be appropriate for all types of antigens to induce CTL responses and that the efficiency of CpG ODNs may be correlated with the mechanism of antigen internalization.

In summary, we demonstrated that CpG ODNs induce different levels of CTL responses and anti-tumor responses when combined with different modified peptide immunizations. CpG ODNs significantly improved the anti-tumor responses of mice following immunization with a long peptide, which had a similar cellular trafficking pathway. However, the anti-tumor responses of mice were limited following immunization with a monolipopeptide, which had a different cellular trafficking pathway. These results are important for the future application of CpG ODN adjuvant in different vaccines.

## Methods

### Cell lines and medium

The TC-1 mouse epithelial cancer cell line, which expresses HPVE6 and E7, was a kind gift from Dr. T-C. Wu (Johns Hopkins University, USA). TC-1 cells were cultured in Dulbecco’s Modified Eagle’s medium (DMEM) (GIBCO-BRL, NY, USA); the media were supplemented with 10% heat-inactivated fetal bovine serum (HyClone, Logan, Utah, USA), penicillin (100 U/ml) and streptomycin (100 μg/ml) (GIBCO-BRL, NY, USA). Complete RPMI-10 medium contained RPMI-1640, which was supplemented with 10% (v/v) heat-inactivated fetal calf serum, 25 mM HEPES (Biological industries, Beit Haemek, Israel), 100 units/ml penicillin, 100 μg/ml streptomycin sulfates and 50 μM β-mercaptoethanol (Sigma, MO, USA).

### Peptide synthesis

Peptides and lipopeptides (Table 1) that contained an MHC-restricted CTL epitope, which was derived from the HPV16 E7 protein or the OVA protein, were purchased from GeneDireX (Nevada, USA). The purity was >90% for all of the peptides. For the flow cytometry and confocal microscopy analyses, free amino groups of peptides and lipopeptides, which were labeled with fluorescein isothiocyanate (FITC), were purchased from GeneDireX (Nevada, USA). The peptides were dissolved in dimethyl sulfoxide (DMSO) (Sigma, MO, USA) at a concentration of 10 mg/ml and stored at −80 °C until use.

### Animals

Female C57BL/6 mice, 6–12 weeks of age, were obtained from the National Laboratory Animal Breeding and Research Center (Taipei, Taiwan). TLR9 knockout (KO) and MyD88 KO mice were purchased from Oriental Bioservice (Tokyo, Japan). All of the animals were housed at the Animal Center of the National Health Research Institutes (NHRI). All of the animal studies were conducted in accordance with the protocol approved by the Institutional Animal Care and Use Committee of the NHRI.

### Culture of BMDCs

BMDCs were harvested as previously described^54^. Briefly, BM cells from the C57BL/6 mice, the TLR9 KO mice and the MyD88 KO mice were cultured at a density of 2 × 10^5^ cells/ml in petri dishes that contained 10 ml of complete RPMI-1640 medium with 200 U/ml (20 ng/ml) recombinant mouse granulocyte-macrophage colony-stimulating factor (GM-CSF; PeproTech Inc., New Jersey, USA). On day 3, another 10 ml of complete RPMI medium that contained 20 ng/ml GM-CSF was added. On day 6, cells from each dish were collected, washed and counted.

### Internalization of peptides

BMDCs were incubated at 37 °C in complete culture medium with FITC-conjugated peptides (IDG) or lipopeptides (lipoIDG) (1 μg/ml) combined with or without 10 μg/ml CpG ODN 1826 (GeneDireX, Nevada, USA). At different time points, the cells were harvested for intracellular fluorescence analysis. The harvested DCs were stained with a PE-labeled anti-CD11c antibody (Ab) (eBioscience San Diego, CA, USA) for 1 h after blocking Fc receptors using an anti-mouse CD16/CD32 Ab (BD mouse Fc-block™). The internalization of the peptides and lipopeptides by CD11c^+^ cells was analyzed using a FACSCalibur flow cytometer (BD Biosciences, USA). In the cell samples, surface-associated fluorescence was quenched using a method that was adapted from Busetto *et al.*^55^, which involved adding an equal volume of 0.1 M citrate buffer (pH 4.0) that contained 250 g/ml trypan blue (Biological Industries, Israel) to each sample and incubating the samples for 1 min on ice before the flow cytometry analysis. Dead cells were gated out by staining with 1 μg/ml propidium iodide.

### Confocal microscopy

To assess the internalization and endocytosis of the peptides, the lipopeptides and CpG ODNs, wild-type (WT) or TLR9 KO BMDCs were seeded at a density of 1 × 10^6^ cells/well in 24-well plates in 1 ml of complete RPMI medium. The cells were incubated with FITC-labeled IDG or lipoIDG combined with or without CpG ODNs or Cy5-conjugated CpG ODNs for 10 or 30 min at 37 °C. After incubation, the cells were fixed in 4% paraformaldehyde (Sigma, MO, USA) and permeabilized in 0.1% NP-40 (Sigma, MO, USA)/PBS. After blocking with 3% bovine serum albumin (BSA) (Sigma, MO, USA)/PBS, the cells were stained with an anti-EEA1 Ab (Abcam, Cambridge, UK) and an Alexa Fluor 568-conjugated goat anti-rabbit IgG Ab (Invitrogen, Oregon, USA) to detect early endosomes. The cells were visualized using a Leica TCS SP5 II confocal microscope (Leitz, Heidelberg, Germany).

### Activation of BMDCs

To investigate the effect of the peptides and lipopeptides on the functional maturation of DCs, 1 × 10^6^ WT BMDCs, TLR9 KO or MyD88 KO BMDCs per ml were plated in complete RPMI-1640 medium. The peptides or lipopeptides (1 μM) combined with or without CpG ODNs (1 μM) were added, and the cells were further incubated for 18 h at 37 °C and 5% CO_2_. As a positive control, the cells were incubated with either 0.1 μg/ml lipopolysaccharide (LPS) or 1 μM CpG ODNs. After incubation, the cells were harvested. The surface markers of DCs were stained with PE-CD11c and APC-CD40 monoclonal antibodies (eBioscience San Diego, CA, USA), and the expression of these makers was analyzed using the FACSCalibur instrument and CellQuest software (BD Bioscience, San Diego, CA, USA). The supernatants of the cells that were cultured for 18 h were isolated and assayed for IL-12p70, IL-6 and TNF-α using a DuoSet ELISA kit (R&D Systems, MN, USA) according to the manufacturer’s protocol.

### Detection of peptide-specific CD8^+^ T cells

C57BL/6 mice were immunized subcutaneously (s.c.) once with 1 μg of peptide mixed with or without 10 μg of CpG adjuvant. After one week, splenocytes were harvested, and the response of IFN-γ-secreting cells was determined by ELISPOT after 48 h of peptide stimulation. Briefly, 2 × 10^5^ splenocytes were incubated with 1 μg/ml irrelevant peptide or RAH peptide in an anti-IFN-γ-coated polyvinylidene fluoride (PVDF) plate for 48 h. After incubation, the cells were removed, and a biotinylated anti-IFN-γ Ab (eBioscience, San Diego, CA, USA) was added to each well. The plates were incubated at 37 °C for 2 h. Following the addition of the avidin-HRP reagent (eBioscience, CA, USA), the assay was developed using a 3-amine-9-ethyl carbazole (AEC; Sigma-Aldrich, MO, USA) staining solution. The reaction was stopped after 4–6 min by placing the plate under tap water. The spots were counted using an ELISPOT reader (Cellular Technology Ltd., Shaker Heights, OH, USA).

For RAH-specific T cell staining, spleens were harvested seven days after the immunizations, and RAH-specific CD8^+^ T cells were detected by tetramer staining using a PE-labeled RAH tetramer (Beckman Coulter, CA, USA) and a FITC-labeled anti-CD8 monoclonal antibody (mAb) (eBioscience, CA, USA). The stained RAH-specific CD8^+^ T cells were analyzed by flow cytometry.

### *In vivo* cytolysis assay

The C57BL/6 mice were single injected with different vaccines (peptides mixed with or without CpG ODNs). After 7 days, 5-carboxyfluorescein diacetate succinimidyl ester (CFSE)-labeled target cells were adoptively transferred to the mice. The target cells were prepared from syngeneic splenocytes that were treated with red blood cell (RBC) lysis buffer (BioLegend) for 2 minutes to remove RBCs, and the cells were subsequently divided into two populations. Each population was pulsed with either 5 μg/ml irrelevant peptide or RAH peptide for 30 min at 37 °C. After the splenocytes were washed in PBS, irrelevant and RAH peptide-pulsed cells were labeled at a final concentration of 1 μM or 10 μM of CFSE (Molecular Probes, Eugene, OR, USA) for 15 minutes at 37 °C. The splenocytes were added to ice-cold complete RPMI medium to stop CFSE labeling. The irrelevant and RAH peptide-pulsed splenocytes were re-suspended in PBS and remixed at a ratio of 1:1. Then, 2 × 10^7^ CFSE-labeled cells were adoptively transferred via tail vein injection into the immunized mice. All of the experimental cells were harvested 18 h after adoptive transfer and analyzed using the FACSCalibur flow cytometer (Becton Dickinson Immunocytometry Systems, San Jose, CA, USA).

### Animal studies

To assess the therapeutic value of the vaccines, tumors were first generated by injecting 2 × 10^5^ TC-1 cells into the abdominal region of the mice. Seven days later, the mice were s.c. vaccinated in a separate abdominal region with 1 μg of non-lipidated peptide (IDG) or lipopeptide (lipoIDG/lipoEQL) mixed with or without 10 μg of CpG ODNs at 200 μl/dose. The tumor volumes were monitored over a 50-day post-tumor implantation period and were calculated according to the following formula: (length × width^2^)/2.

## Additional Information

**How to cite this article**: Song, Y.-C. and Liu, S.-J. A TLR9 agonist enhances the anti-tumor immunity of peptide and lipopeptide vaccines via different mechanisms. *Sci. Rep.* **5**, 12578; doi: 10.1038/srep12578 (2015).

## Supplementary Material

Supplementary Information

## Figures and Tables

**Table 1:** Peptides and lipopeptide containing MHC class I-restricted CTL epitope derived from HPV16 E7 protein and OVA protein are used in this study.

**Peptide name**	**Peptide composition**		
	**lipid**	**spacer sequence**	**peptide sequence**
RAH			RAHYNIVTF
IDG			IDGPAGQAEPDRAHYNIVTFCCKC
lipoIDG	Palmitic acid	KSS	IDGPAGQAEPDRAHYNIVTFCCKC
lipoEQL	Palmitic acid	KSS	EQLESIINFEKLTEWTSS

— The bottom-line is the sequence of MHC class I-restricted CTL epitope.
