# Supplementary material for: A TLR9 agonist enhances the anti-tumor immunity of peptide and lipopeptide vaccines via different mechanisms
Source: Sci Rep. 2015 Jul 28;5:12578. doi: 10.1038/srep12578 (PMC4517169; doi:10.1038/srep12578)
Supplement: Supplementary Information [file srep12578-s1.pdf]

A TLR9 agonist enhances the anti-tumor immunity of peptide and lipopeptide vaccines via different mechanisms

Ying-Chyi Song and Shih-Jen Liu

Supplementary Fig. 1

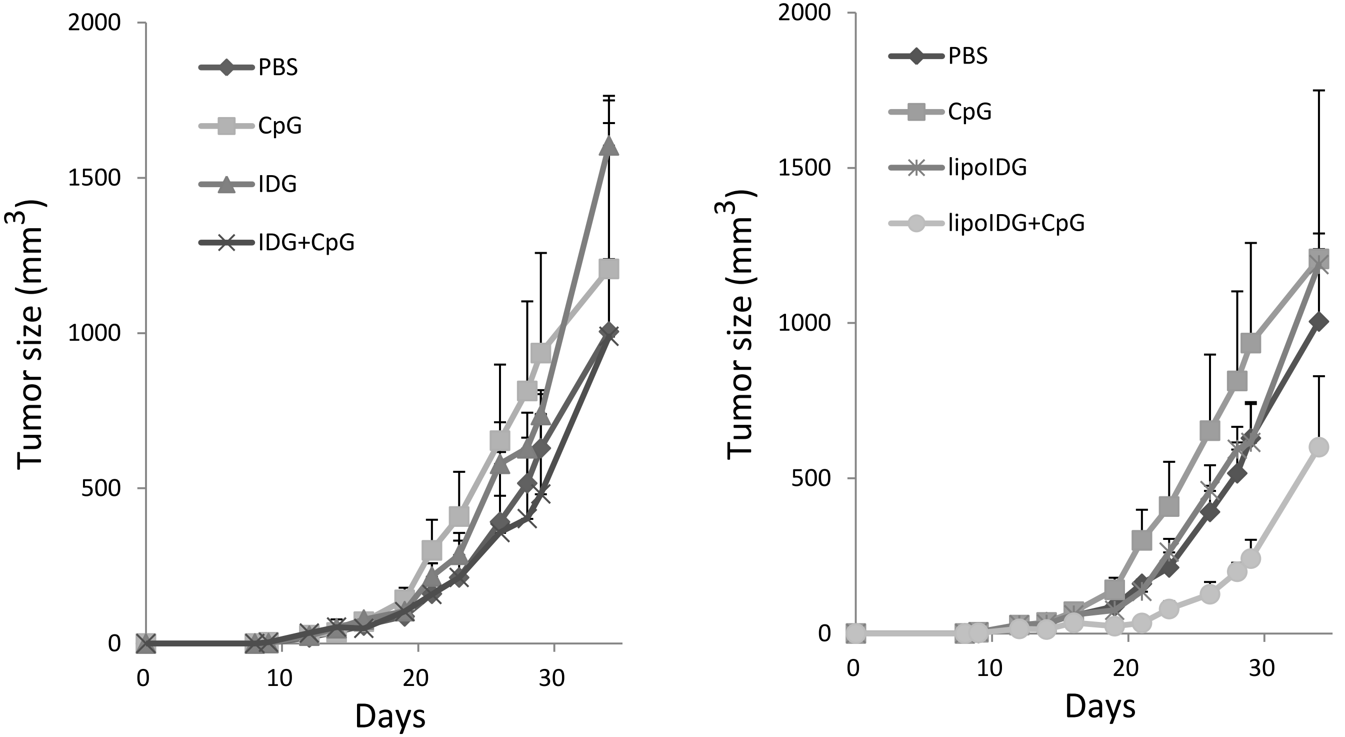

Supplementary Fig. 2

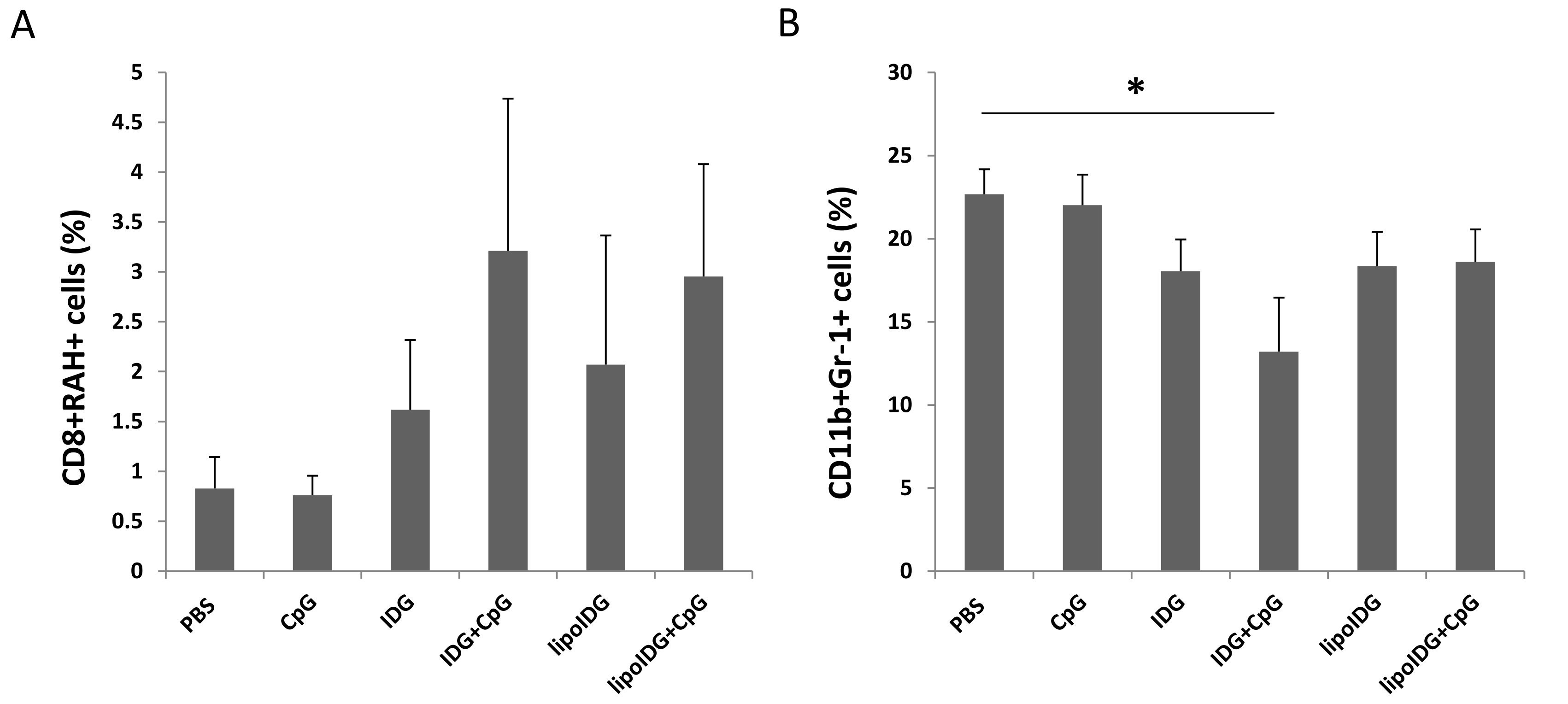

Supplementary Fig. 3

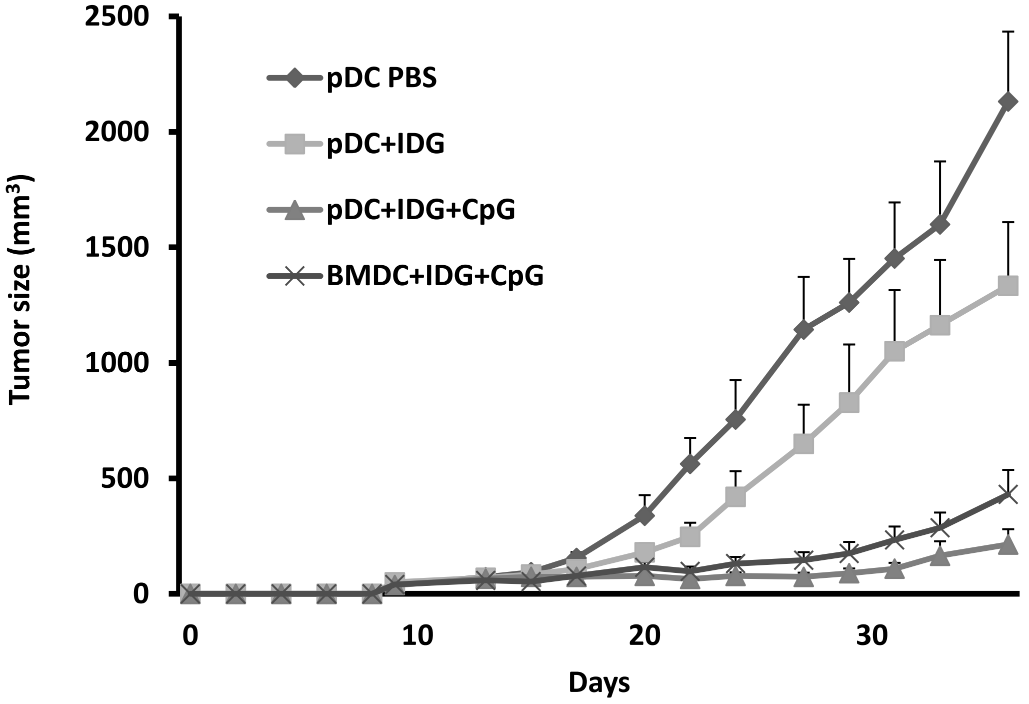

Supplementary Fig. 4

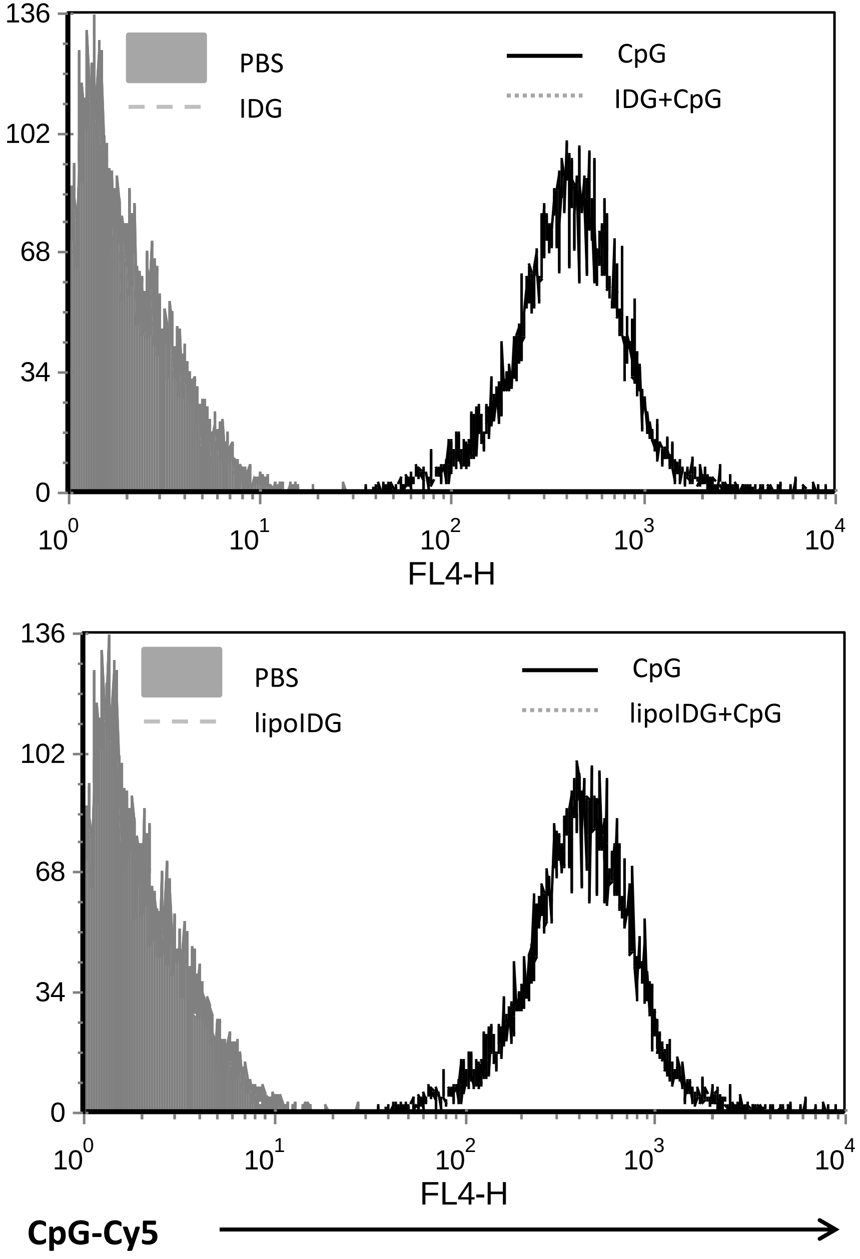

Supplementary Figure 1 | CpG ODN adjuvant induces anti-tumor effects, which are reversed in MyD88 KO mice. TC-1 tumor-bearing MyD88 knockout (KO) mice (3 animals/group) received a single injection with IDG or lipoIDG (1 μg) admixed with or without CpG ODNs (10 μg). PBS or CpG alone was used as a control. The tumor diameters are shown (mm^3). The data are expressed as the means + SEM.

Supplementary Figure 2 | Analysis of tumor-infiltratin leukocytes. Tumor-bearing mice were immunized with IDG or lipoIDG (1 μg) mixed with or without CpG ODNs (10 μg) 7 days post-tumor cell implantation. After immunization for 8 days, the tumors removed from the tumor-bearing mice were minced and passed through a 70-μm filter, and the all of the cells were stained with anti-CD45 Ab to quantify tumor-infiltrating leukocytes, anti-CD8 Ab and RAH-tetramer to quantify antigen specific cytotoxic T lymphocytes (CTLs), and anti-CD11b Ab and anti-Gr-1 Ab to quantify myeloid-derived suppressor cells (MDSCs). The data represent the percentage of the indicated markers in tumor-infiltrating leukocytes. The data are expressed as the means + SEM.

Supplementary Figure 3 | Peptide-pulsed BMDCs/pDCs induce anti-tumor effects *in vivo*. The bone marrow (BM) cells from the C57BL/6 mice were cultured with 200 U/ml (20 ng/ml) GM-CSF for 6 days (BMDCs) or 100 ng/ml Flt3L for 9 days (pDCs). The cultured DCs (BMDCs/pDCs) (1×10^6 cells/ml) were pulsed with PBS or IDG (1 μg) combined with or without CpG ODNs (10 μg) in RPMI-1640 medium at 37°C for 20 h. After washing, 100 μl of the BMDC/pDC suspension (2×10^5 cells) was injected i.v. into TC-1 tumor-bearing mice. The tumor diameters were measured and calculated from the measurements according to the following formula: $(\text{length} \times \text{width}^2)/2$.

Supplementary Figure 4 | Internalization efficiency of CpG ODNs combined with peptides and lipopeptides. The cellular internalization activity of CpG ODNs was determined by flow cytometry after treating DCs with FITC-conjugated IDG or lipoIDG (1 μg/ml) combined with or without Cy5-conjugated CpG ODNs (10 μg/ml) for 120 min. After trypan blue quenching, the internalization of the peptides and lipopeptides by $CD11c^+$ cells was analyzed by flow cytometry. Dead cells were gated out by propidium iodide.
